# Determination of Hyaluronic Acid Dermal Filler Impurities Using SEM/EDS Analysis

**DOI:** 10.3390/polym15071649

**Published:** 2023-03-26

**Authors:** Won Lee, Nark-Kyoung Rho, Eun-Jung Yang

**Affiliations:** 1 Yonsei E1 Plastic Surgery Clinic, Anyang 14072, Republic of Korea; 2 Department of Dermatology, Sungkyunkwan University School of Medicine, Suwon 16419, Republic of Korea; 3 Leaders Aesthetic Laser & Cosmetic Surgery Center, Seoul 06014, Republic of Korea; 4 Department of Plastic and Reconstructive Surgery, Yonsei University College of Medicine, Seoul 03721, Republic of Korea

**Keywords:** hyaluronic acid filler, impurities, filler complications, scanning electron microscopy

## Abstract

Although hyaluronic acid (HA) filler injections are associated with various non-vascular inflammatory complications, the underlying mode of action remains unclear. The hyaluronic acid filler may not be sufficiently pure, leading to an immune response. The present study attempted to identify any impurities in hyaluronic acid fillers available on the market. Particles were counted after degrading hyaluronic acid filler with hyaluronidase. Particulate matter was subsequently observed using scanning electron microscopy, and the particle components were evaluated using energy-dispersive X-ray spectroscopy. Different quantities of impurity particles (>10 and 25 μm) were detected microscopically. Silicon and aluminum isotopes were also detected. Hyaluronic acid fillers were contaminated with these particles. The degree of contamination varied substantially among the tested filler products. These contaminant particles may evoke reactions in the patient’s body. Clinicians should be aware of this source of possible contamination and its effects.

## 1. Introduction

Hyaluronic acid (HA) filler injections are among the most commonly performed aesthetic procedures [1]. This procedure is relatively easy to perform and yields rapid results. HA fillers can be manufactured using various processes [2]. Generally, fillers are developed using HA powder and a crosslinker such as 1,4-butanediol diglycidyl ether (BDDE) [3]. Crosslinking using BDDE prevents the enzymatic degradation of HA and increases its stability. These modifications result in a diverse set of HA fillers with different cohesive and viscoelastic properties. The manufacturing processes of diverse HA filler products differ based on temperature, amount of crosslinker used, dialysis methods, and washing processes [4].

With the increasing use of HA fillers, the associated complications have also increased. HA-filler-related complications can be divided into vascular and non-vascular types [5]. Vascular complications are relatively severe problems related to vascular anatomy. Non-vascular complications include nodules, granulomas, and delayed-type hypersensitivity. The etiology of non-vascular complications is multifactorial, and several hypotheses have been proposed. Glycosaminoglycans, such as HA, have been reported to act as superantigens and directly trigger immune responses [6]. Others have posited a mechanism by which the breakdown of HA gels into low-molecular-weight HA fragments with proinflammatory properties can trigger a systemic inflammatory response [7]. A recent publication postulated that an influenza infection or the introduction of a medication, such as an antibiotic, antipyretic, or non-steroidal anti-inflammatory drug, against infectious agents following an HA injection can initiate a chain of events, leading to delayed hypersensitivity reactions by activating T lymphocytes through CD4+ cells [8]. Recently, several patients vaccinated against coronavirus disease have reported post-filler-injection inflammation owing to delayed hypersensitivity [9].

HA fillers produced through various manufacturing processes may also have issues with injectable materials. Theoretically, the products should consist only of biocompatible HA and a crosslinker. However, obtaining injectable drug products that are free of chemicals or particulate matter contamination remains challenging [10]. Owing to various manufacturing processes, impurities may be introduced, causing non-vascular-inflammatory adverse effects in patients receiving injections. Although HA fillers are also injectable, little is known about HA filler impurities. In this study, the impurities present in HA filler products available on the market were examined. To the best of our knowledge, this is the first study to perform such an examination.

## 2. Materials and Methods

### 2.1. HA Dermal Filler

Twelve HA filler products were tested, namely, Restylane Volume (LOT 18684-1, Galderma, Uppsala, Sweden), Juvéderm Voluma (LOT VB20A90809, Allergan, Irvine, CA, USA), Belotero Volume (LOT 547253/1, Merz Pharmaceuticals, Frankfurt, Germany), Teosyal RHA4 (LOT TPUL-183815C, TEOXANE Laboratories, Geneva, Switzerland), L’orient No. 6 (LOT J20001, Joonghun Pharmaceutical, Seoul, Republic of Korea), Neuramis Volume (LOT C521007A, Medy-Tox, Seoul, Republic of Korea), The Chaeum Premium No. 3 (LOT BLD21008, HUGEL, Seoul, Republic of Korea), Elravie Deep (LOT D1915001BA, Humedix, Seoul, Republic of Korea), Eptq S500 (LOT YLC18012, Jetema, Seoul, Republic of Korea), QTfill SubQ (QPAI21001G, S.THEPHARM, Seoul, Republic of Korea), Youthfill Deep (LOT YDE19019, RFBio, Seoul, Republic of Korea), and Cleviel Contour (LOT J94118001, PharmaResearch, Seongnam, Republic of Korea). The lot number of each product was recorded, and the corresponding results were described by randomly assigning products with a letter, such as A, B, and C, for identification. A minimum of two 1 mL fillers were prepared for each product for use in particle analysis and scanning electron microscopy (SEM).

### 2.2. Hyaluronidase Preparation

Type I-S (lyophilized powder, approximately 400–1000 units/mg solid, Sigma-Aldrich, St. Louis, MO, USA) hyaluronidase from bovine testes was used, with an average activity of 748 units/mg. A total of 13.36 mg of hyaluronidase powder was mixed with 20 mL of purified water to obtain a concentration of 10,000 IU/mL. After dissolving the powder completely, a 0.22 μm syringe filter was used to filter the solution to remove any existing particles of hyaluronidase powder.

### 2.3. HA Filler Degradation

Hyaluronidase (2 mL; 20,000 IU) solution was prepared in a 50 mL tube and mixed with HA filler (1 mL). Filtered purified water (17 mL) was added to the 5 mL tube to obtain a total volume of 20 mL, as 50 mL of the solution was required for insoluble particle analysis. The syringe was placed in a 37 °C water tank, and the tank was shaken for 24 h to degrade the HA filler completely. Although there were a few differences in the degradation times of different HA filler products, 24 h of shaking in a warm water tank was sufficient for HA filler degradation, as described in previous studies [11,12].

### 2.4. Insoluble Particle Analysis

**Figure 1:**
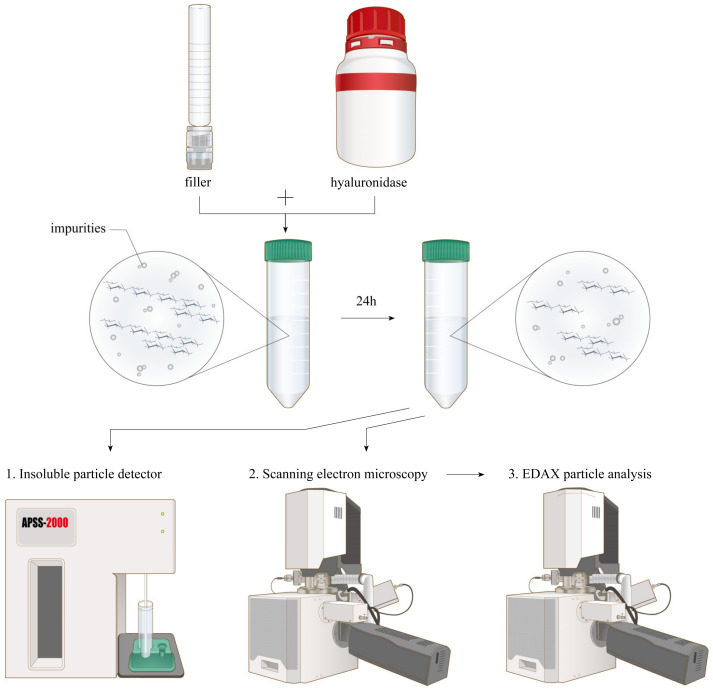
A representation of the impurity particle analysis process.

Each sample was placed in an insoluble particle counter (APSS-2000; Automated Parenteral Sampling System, Particle Measuring Systems, Boulder, CO, USA). Hyaluronidase alone was used as a negative control. Four tests were performed, and a 5 mL sample was used for each insoluble particle analysis test. The first test was excluded to avoid bias. Particles >10 and 25 μm, the sizes defined by the United States Pharmacopeia (USP) for particulate matter in injections [8], were counted. The average results of the three tests were recorded. Details of the analysis are presented in Figure 1.

Hyaluronic acid filler was degraded for 24 h with hyaluronidase. No visible hyaluronic acid filler particles were detected after degradation. The insoluble particles were counted, visualized using scanning electron microscopy, and analyzed using energy-dispersive X-ray spectrometry.

At a magnification of 350×, 1 or 0 detected insoluble particles were classified as “very few,” and at a magnification of 500×, approximately 1–5 and <5 particles were classified as “few” and “many,” respectively.

### 2.5. SEM Analysis

HA filler (1 mL) was prepared and degraded with hyaluronidase following the procedure described in ‘Hyaluronidase Acid Filler Degradation.’ Degraded fillers were poured onto filter paper (pore size 1 μm). The residue was analyzed using SEM (2 °C, 747 Pa, 100% humidity, Quattro S SEM, approximately 250–5000×, Thermo Fisher, Waltham, MA, USA) at the Korea Advanced Institute of Science and Technology.

### 2.6. Energy-Dispersive X-ray Spectrometry (EDS) Analysis

The impurities detected during SEM analysis were evaluated using EDS analysis. EDS is a chemical microanalysis technique performed in conjunction with SEM. HA filler (1 mL) was prepared and degraded using hyaluronidase following the procedure described in ‘Hyaluronidase Acid Filler Degradation.’ Degraded fillers were poured onto filter paper (pore size 1 μm). A targeted X-ray beam was used to detect the isotopes of the impurities using EDS (Quattro S SEM, Thermo Fisher, Waltham, MA, USA) at the Korea Advanced Institute of Science and Technology.

## 3. Results

### 3.1. Insoluble Particle Analysis

Twelve HA filler products with variable counts of insoluble particles were evaluated (Table 1). Product C showed the lowest average particle count, in which 1033 particles >10 μm were present. In contrast, product I had an average of 119,856 particles >10 μm. Product D had the least detected particles >25 μm (average: 17), whereas product I had the most (average: 6254). In the control, an average of 37 and 12 particles >10 μm and >25 μm, respectively, were observed.

### 3.2. SEM Analysis

**Figure 2:**
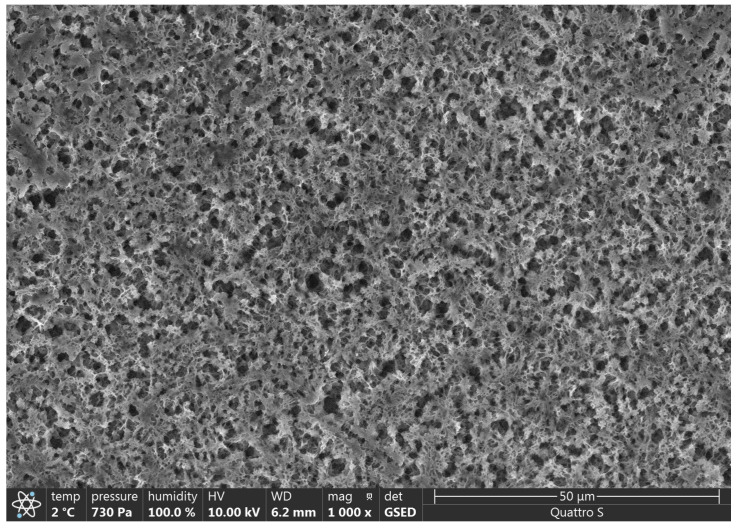
Illustration showing the magnification of Product E (1000×). Impurity particles were not observed.

**Figure 3:**
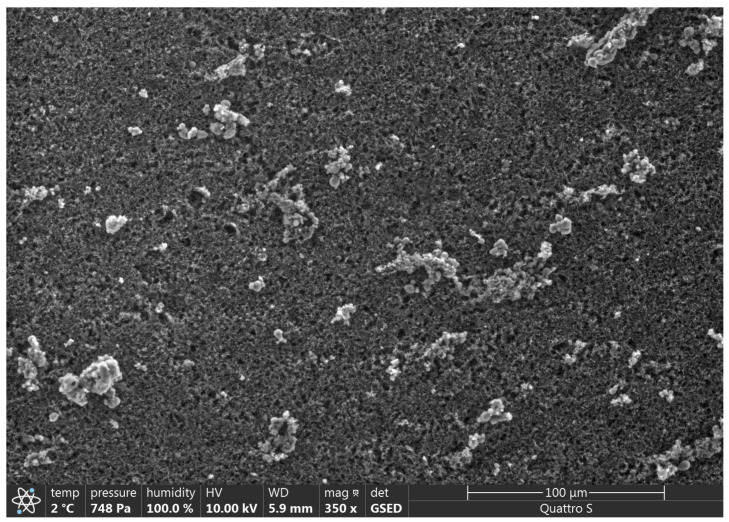
Illustration showing the magnification of Product K (350×). Multiple impurity particles were observed.

**Figure 4:**
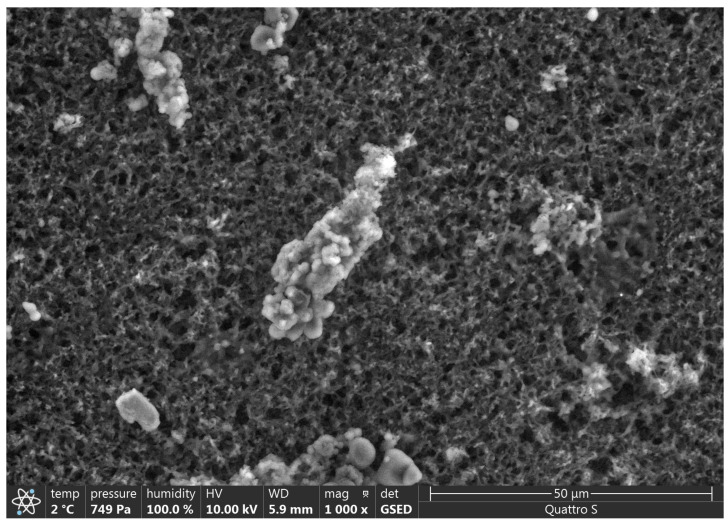
Illustration showing the magnification of Product K (1000×). A particle of >50 μm was observed.

**Figure 5:**
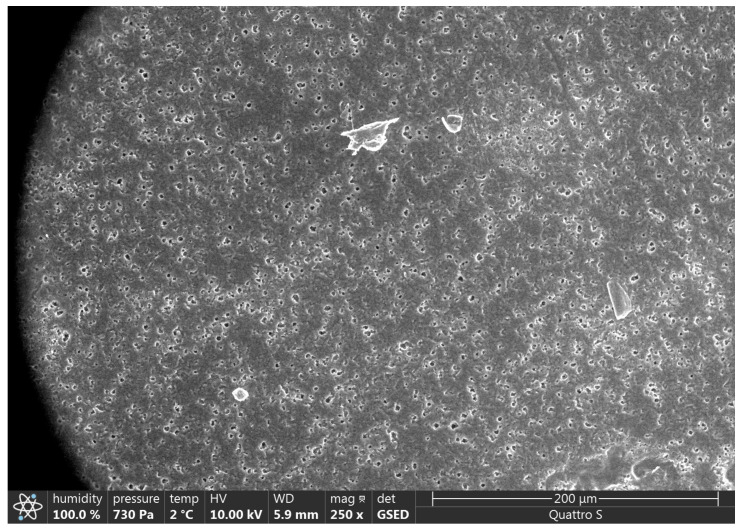
Illustration showing the magnification of Product G (250×). Multiple insoluble particles were observed.

**Figure 6:**
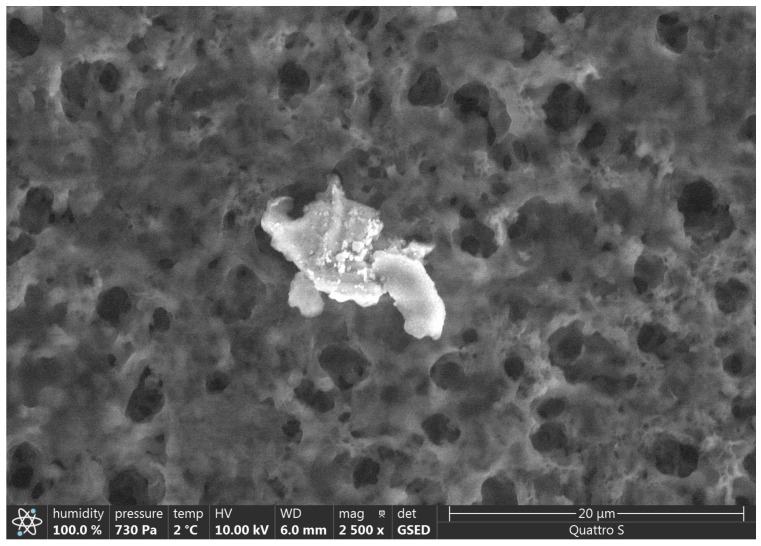
Illustration showing the magnification of Product J (2500×). A particle of >10 μm was observed.

**Figure 7:**
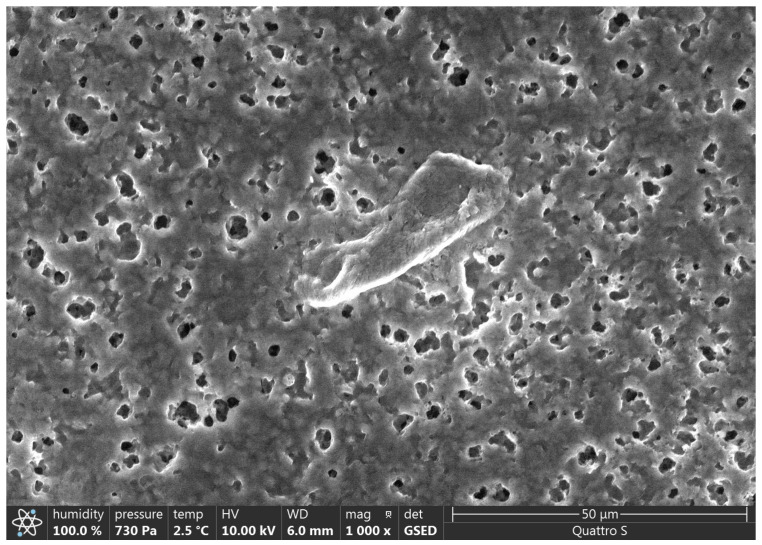
Illustration showing the magnification of Product L (1000×). A particle of >50 μm was observed.

SEM results are presented in Figure 2, Figure 3, Figure 4, Figure 5, Figure 6 and Figure 7.

Various impurities were detected in these products (Table 2). Multiple impurity particles were observed in product K at 350× magnification. At 1000× magnification, particles >50 μm were observed. Multiple impurities were detected in products G, J, and L.

### 3.3. EDS Analysis

**Figure 8:**
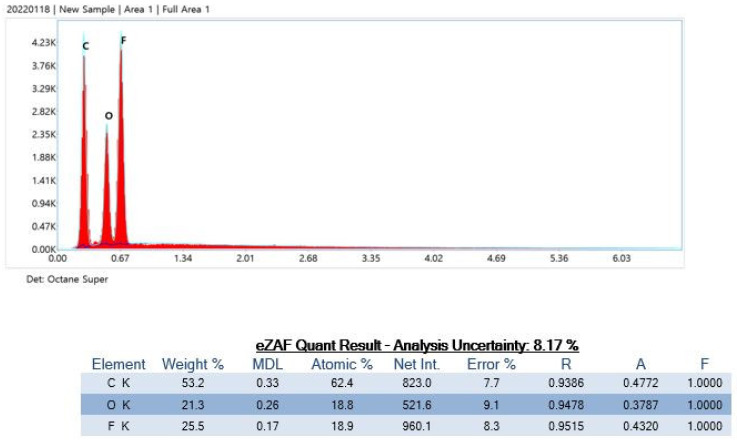
Particle analysis using energy-dispersive X-ray spectrometry.

After the impurity particles were detected using SEM, a targeted X-ray beam was used to detect the isotopes of the impurities (Table 2). Product E contained C, O, and F isotopes, and normal filter paper isotopes (Figure 8).

The particles from Product E were C, O, and F isotopes, which are normal filter paper isotopes (atomic: mole ratio, Net Int.: real valid data, error %: error percentage <30% can be determined for data with confidence, A: absorbance, F: spectroscopy).

**Figure 9:**
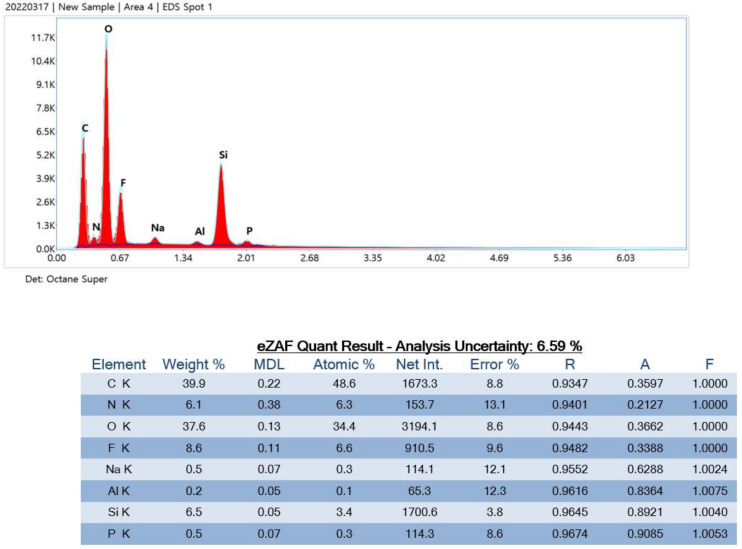
Particle analysis using energy-dispersive X-ray spectrometry.

The contaminants detected in product G contained Si isotopes, which may have been incorporated during the manufacturing process (Figure 9).

Product G particles contained the Si isotope.

**Figure 10:**
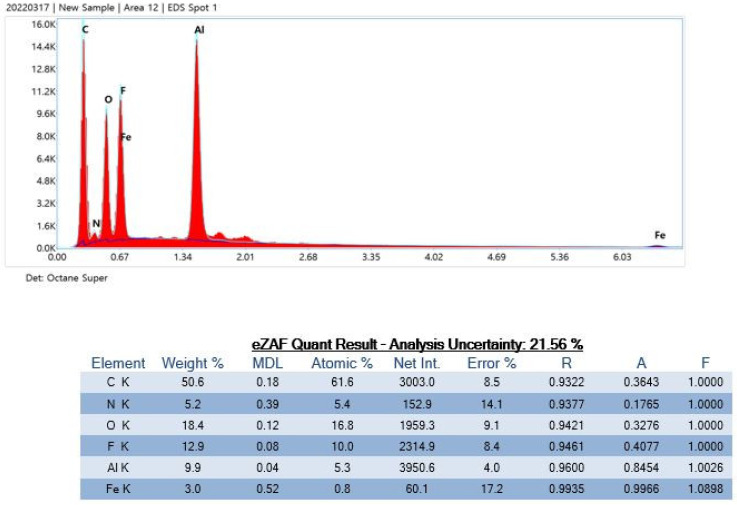
Particle analysis using energy-dispersive X-ray spectrometry.

Product J contained Al isotopes, which may have been incorporated during the manufacturing process (Figure 10).

Product J particles contained Al and Fe isotopes.

**Figure 11:**
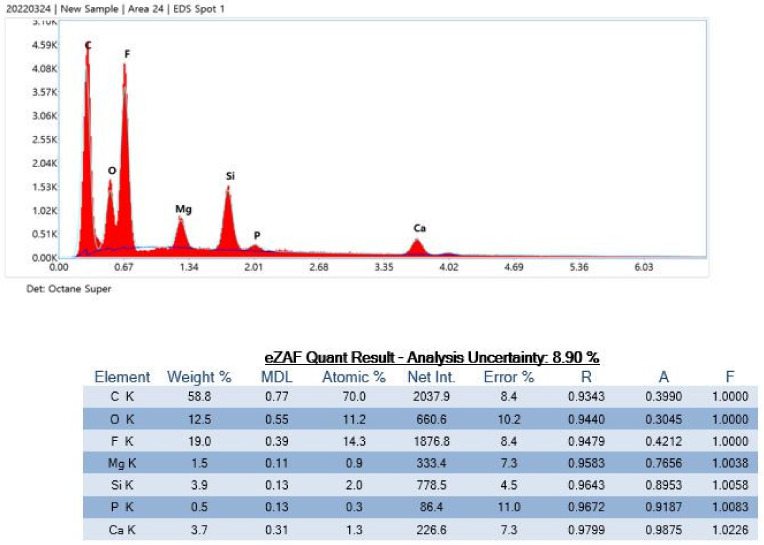
Particle analysis using energy-dispersive X-ray spectrometry.

Product D contained Si, Mg, and Ca isotopes (Figure 11).

Product D particles contained Si, Mg, and Ca isotopes.

**Figure 12:**
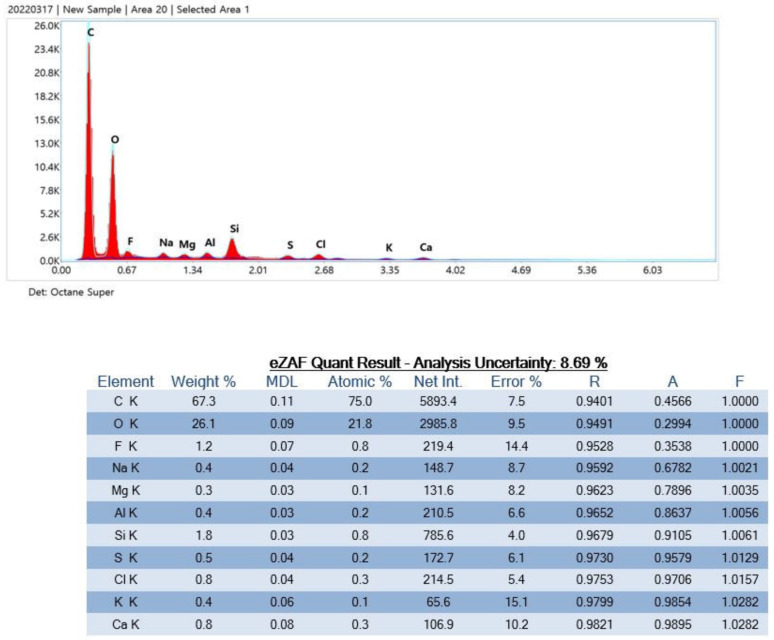
Particle analysis using energy-dispersive X-ray spectrometry. Product F particles contained Si, Al, Mg, and Ca isotopes.

Product F contained Si, Al, Mg, and Ca isotopes (Figure 12). As the number of particles in the control particle analysis was too low, EDS analysis could not be performed.

## 4. Discussion

Several studies have investigated HA filler-related complications; however, the causes of the resultant non-vascular complications have not been fully identified. It was hypothesized that the HA filler may not be sufficiently pure owing to its production using multiple manufacturing processes, and that these contaminated HA fillers may cause immune reactions in patients. In this study, an attempt was made to identify any impurities in hyaluronic acid fillers available on the market. Particles were counted after degrading the HA filler with hyaluronidase. The particulate matter was subsequently observed using SEM, and the particle components were evaluated using EDS. Different quantities of impurity particles (>10 and 25 μm) were detected microscopically. Silicon and aluminum isotopes were also detected. It was found that HA fillers were contaminated with these particles. The degree of contamination varied substantially among the tested filler products.

The USP defines particulate matter as mobile, undissolved particles, other than gas bubbles, that are unintentionally present in a solution [13]. Additionally, particulate matter is commonly categorized as visible (>100 μm) or subvisible [8]. Several studies have been conducted on this subject; however, they primarily investigated particulate contaminants in intravenous infusions, and most were conducted in the 1970s and 1980s [14]. With the rapidly growing class of protein drugs in use, clinicians have expressed a renewed interest in this topic, focusing on the impact of particles on patients. However, there has been little discussion regarding aesthetic medicine and surgery.

Particulates can be classified as extrinsic, intrinsic, or inherent (Table 3).

**Figure 13:**
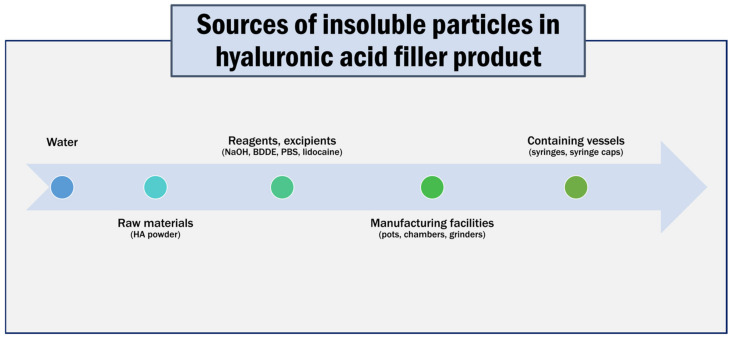
Common sources of particulates.

Extrinsic particulate matter is derived from foreign or unexpected sources and not from product formulation, packaging, or processing. Intrinsic particulate matter is material that arises from product formulation, packaging, and processing sources. Inherent particulate matter is material expected to originate from product formulations and can be accepted as a product characteristic [15]. All injectable products are contaminated with various amounts of particulate matter. These insoluble particles enter the products primarily during the manufacturing process (Figure 13) [10].

Common sources of particulates during the manufacture of a hyaluronic acid filler product can result in additive accumulation.

One of the most common types of metal found in liquid-injectable pharmaceuticals is stainless steel, which is thought to be derived from the machinery used during the manufacturing processes [10]. The presence of stainless-steel particles is a frequent cause of recall for injectable lipid emulsion products, such as propofol [16]. For injectable fillers, the prefilled syringe fabrication process is viewed as a potential hazard for metal particle contamination [17]. Ferrochromium and stainless-steel alloys are characterized by chromium- and iron-rich surfaces and additional alloying elements, such as manganese, molybdenum, and nickel, which are present in variable amounts [18]. Contamination of injectable products with ultrafine stainless-steel particles can be hazardous because chromium is among the most dangerous metal contaminants found in injectables [10].

Prefilled glass syringes are standard containers used for most injectable filler products. The risk of Al particle contamination was relatively high when the prefilled syringe barrel was composed of type I borosilicate glass containing 5% aluminum oxide. Al particles can form when glass delamination occurs during the manufacturing and handling of prefilled glass syringes. Furthermore, they can leach from glass barrels and rubber stoppers during storage [19,20]. Persistent dermal or subcutaneous nodules have been identified as injection-site complications for aluminum-adsorbed vaccines and depot injections [21,22].

In contrast with foreign-body granulomas, injection-site granulomas histologically show a distinct lymphocytic infiltrate with nodular lymphoid aggregation and abundant eosinophils, consistent with a delayed granulomatous hypersensitivity reaction secondary to the persistence of aluminum crystals [22]. Although Al does not induce cellular immune responses, it can induce an enhanced humoral immune response [23]. The adsorption of specific antigens onto aluminum salts may result in a high local antigen concentration at the injection site and enhanced uptake by antigen-presenting cells. Aluminum particles can further enhance the immune response via direct or indirect stimulation of dendritic cells by activating the complement system and inducing chemokine release [24].

Although solid Si implants are commonly used as medical implants and are considered inert, Si oil microdroplets from prefilled syringes may cause delayed inflammatory responses. Si oil, which is added to most commercial syringes as a lubricant, can contaminate injected materials and lead to inflammatory complications. The total amount of Si oil (~0.6–1.0 mg/mL) and its distribution in prefilled syringes can impact syringe functionality and particle formation [25,26]. The siliconized polypropylene insulin syringes showed significantly higher silicone oil particle counts than the prefilled glass syringes [27,28].

Laboratory studies have shown that Si oil microdroplets can act as adjuvants to promote immunological tolerance and induce an antibody response [29]. Similarly, a recent study suggested a causal link between siliconized syringes and ocular inflammation following intravitreal injection [29]. There are several reports of Si granulomas after direct injection of dermal fillers or secondary leakage from Si breast implants [29]. Cases of sclerosing lipogranulomas resulting from syringe-lubricant-contaminated injections have also been reported [30,31]. The results of a recent study [32] indicated that submicron- and micron-sized Si oil droplets could increase the secretion of macrophage inflammatory protein-1α and proinflammatory cytokines—interleukin-6, interleukin-8, and tumor necrosis factor—in human peripheral blood mononuclear cells, indicating that Si oil droplets can potentially evoke early and late immune responses. Si oil, a well-known molecule that causes autoimmune responses, may augment the delayed inflammatory responses caused by HA fillers [29]. Healthcare professionals should be aware of the use of Si oil in syringe manufacturing, factors that promote its release, and potential complications after dermal filler injections. Given the risks associated with Si oil immunoreactivity and the lack of an elimination mechanism, prefilled syringes that use less Si oil during the manufacture of injectable filler products should be considered.

Subvisible particles are considered critical quality attributes in biopharmaceutical manufacturing. According to the USP [13] and the European Pharmacopeia [33], the thresholds for particle levels in a standard injectable drug in a prefilled syringe are 6000 and 600 per container for particles ≥10 and ≥25 μm, respectively. However, in most countries, these standards do not apply to dermal fillers as they are registered as medical devices rather than drugs. No limits or standards specific to particulate matter in injectable filler products have been defined. Assuming that high-purity HA raw materials are processed, these impurities are thought to occur during manufacturing; however, the results of the experiments alone are insufficient to determine the source or reason for the impurities. Although medical-grade lubricants and metals found in dermal filler products may be present in small quantities, they can induce an immune response in the injected tissue, especially when the filler persists for extended periods, and contribute to a small risk of delayed-onset nodules. Currently, there is no information available to clinicians other than individual experimental measurements. In this study, the different Si contents for each manufacturer of injectable fillers were determined. Clinicians should be informed of the particulate matter issue, and the industry should strive to minimize this potential risk while manufacturing medical devices for aesthetic purposes.

The main limitation of this study is that it did not confirm the inflammatory potential of particulate matter in the samples. This study mainly aimed to experimentally confirm these products’ potential to cause non-vascular adverse effects. This experiment showed that there are impurities in commercially available HA fillers. Some fillers had impurity levels that were higher than the permissible limits for injectables, such as common intravenous fluids and drugs. However, the clinical relevance of the results of this study—that is, the association between the impurities and non-vascular side effects—requires future large-scale clinical studies. Based on the preliminary results obtained in this study, which are believed to be the first to describe particulate matter present in HA filler products, further in vitro and animal studies with clinical relevance are recommended. When further validation studies confirm our data, it may be feasible to use EDS as a quality control test to precisely analyze the counts of insoluble particles in injectable HA products.

## 5. Conclusions

HA fillers were found to be contaminated with particles. The degree of contamination varied significantly among the tested filler products. These contaminants can trigger inflammatory immune reactions. Therefore, clinicians should be aware of the possible contamination and the resultant inflammatory immune responses. Most patients that want filler injections are individuals seeking cosmetic treatments to address concerns about their appearance and not diseased patients. Thus, the public demand for the quality control of cosmetic injectables may increase further. To the best of our knowledge, no previous reports have compared insoluble particles in commercial HA filler injectables detected using semi-quantitative analyses, such as SEM/EDS. Understanding the purity of injectable HA fillers ensures the manufacture of the highest-quality products that meet regulatory and consumer expectations for safety and efficacy.

## Figures and Tables

**Table 1:** HA filler products with variable counts of insoluble particles.

	Larger than 10 μm (Count/Syringe)				Larger than 25 μm (Count/Syringe)			
	1st	2nd	3rd	Average	1st	2nd	3rd	Average
A	4048	3012	3528	3529	60	32	72	54
B	1352	1264	1272	1296	156	112	118	128
C	1304	968	828	1033	72	44	28	48
D	3632	3588	3536	3585	28	8	16	17
E	1204	1340	1196	1246	88	72	88	82
F	76,520	79,792	76,588	77,633	812	1044	876	910
G	11,468	10,460	10,804	10,910	312	312	236	286
H	16,264	16,816	17,084	16,721	936	916	936	929
I	121,244	120,184	118,140	119,856	6156	6600	6008	6254
J	27,912	29,712	28,180	28,601	120	84	96	100
K	28,892	25,780	25,480	26,717	896	756	820	824
L	46,408	43,516	40,568	43,497	992	1100	1092	1061
Control	44	32	36	37	20	4	12	12

**Table 2:** Scanning electron microscopy (SEM) and energy-dispersive X-ray spectrometry (EDS) analysis of filler products.

Filler Product	SEM (Particles Detected)	EDS Isotopes
A	Few	C O F Mg
B	Few	C O F Mg P
C	Very few	C O F Mg P
D	Few	C O F Mg Si P Ca
E	Very few	C O F
F	Many	C O F Si Al Mg Ca
G	Few	C O F Si Al
H	Few	C O Si
I	Many	C O F Si Al P
J	Many	C O F Al Fe
K	Many	C O Si
L	Many	C O F Al Si P S Cl K Ca

**Table 3:** Types of particles in the injectables as described in USP [10].

Type	Source	Example
Extrinsic	Contamination from an external source	Dust Particles of biological origin
Intrinsic	Particles derived from contact of the product with materials used in the containers or packaging	Silicone particles Polymeric materials Metal particles Glass particles Rubber particles
Inherent	Particles related to the process - The result of interactions with the manufacturing equipment during the production process - May arise from physical or chemical changes during the production process	Protein aggregates Colloidal formulations Insoluble degradation products’ active ingredients Crystals of active ingredients Metal particles

## Data Availability

Datasets generated and/or analyzed in the current study are available from the corresponding author upon reasonable request.
